# Fingerspelling and Its Role in Translanguaging

**DOI:** 10.3390/languages7040278

**Published:** 2022-11-01

**Authors:** Brittany Lee, Kristen Secora

**Affiliations:** 1 Psychological Sciences, University of Connecticut, Storrs, CT 06269, USA; 2 Theory and Practice in Teacher Education, University of Tennessee Knoxville, Knoxville, TN 37996, USA

**Keywords:** fingerspelling, American Sign Language, translanguaging, deaf readers, bimodal bilingualism

## Abstract

Fingerspelling is a critical component of many sign languages. This manual representation of orthographic code is one key way in which signers engage in translanguaging, drawing from all of their linguistic and semiotic resources to support communication. Translanguaging in bimodal bilinguals is unique because it involves drawing from languages in different modalities, namely a signed language like American Sign Language and a spoken language like English (or its written form). Fingerspelling can be seen as a unique product of the unified linguistic system that translanguaging theories purport, as it blends features of both sign and print. The goals of this paper are twofold: to integrate existing research on fingerspelling in order to characterize it as a cognitive-linguistic phenomenon and to discuss the role of fingerspelling in translanguaging and communication. We will first review and synthesize research from linguistics and cognitive neuroscience to summarize our current understanding of fingerspelling, its production, comprehension, and acquisition. We will then discuss how fingerspelling relates to translanguaging theories and how it can be incorporated into translanguaging practices to support literacy and other communication goals.

## 1. Introduction

Sign languages, like spoken languages, are natural human languages with their own phonology, grammar, and syntax. One unique component of many signed languages is fingerspelling, a natural part of real-time communication in which designated handshapes represent the letters of the surrounding spoken language’s alphabet. For example, American Sign Language (ASL) includes one-handed handshapes with specific orientations that correspond to the letters of the English alphabet. Fingerspelling is a manual representation of orthographic code and is one of the main ways in which Deaf and Hard of Hearing people engage in translanguaging.

Here, we use the term ‘translanguaging’ to refer to a practice whereby an individual leverages all of their languages in order to create meaning (Swanwick 2017). There are a number of studies that report multimodal translanguaging, which involves using different modalities to convey meaning (e.g., speech, writing, gesturing, pointing, use of diagrams, images, graphics) (Holmström and Schönström 2018; Mazak and Herbas-Donoso 2015). However, in these cases, the multiple modalities involve one or more spoken language, one or more written language, pictures/diagrams, and/or physical body movements. For example, Canals (2021) reported on the translanguaging strategies between English- and Spanish-speaking undergraduate students. Speakers used a variety of languages and symbols across different modalities in order to arrive at a shared understanding. Translanguaging strategies were used in approximately half of the episodes in which speakers needed to negotiate meaning by incorporating a variety of tools in addition to spoken languages (e.g., use of devices to show words/images, written notes, gesturing, pointing). Speakers used these strategies for a variety of communication purposes, including requesting clarification, asking follow-up questions, explanation, reformulating responses, providing feedback or lexical items, and clarifying meaning or pronunciation. This study is representative of what the multimodal translanguaging literature for spoken language shows: speakers construct shared meaning using a variety of modalities and strategies, including spoken languages, written language, nonverbal communication (e.g., gestures, pointing, posture), and pictures.

There are far fewer studies investigating translanguaging in deaf-deaf or deaf-hearing communication than between hearing-hearing speakers (e.g., Swanwick 2017; Swanwick and Watson 2007). While all communicators use multimodal translanguaging strategies, translanguaging in bimodal bilinguals is unique because their languages already span two modalities: a signed language like ASL (produced by the hands, face, and body in space) and a spoken language like English (produced by the lips, tongue, jaw, and vocal tract) or its written form. Fingerspelling plays as an especially important role in translanguaging because of its connections to both sign and print, yet it is an understudied topic.

The first goal of this paper is to integrate existing research on fingerspelling in order to characterize it as a cognitive-linguistic phenomenon. In this review, we will provide a brief introduction to fingerspelling from a linguistic perspective, addressing several key questions: What is fingerspelling? What does it look like? What is it used for? How does it differ across signed languages and orthographic systems? We will review and synthesize research to summarize our current understanding of how fingerspelling is produced, comprehended, and acquired, drawing from cognitive neuroscience and studies that employ various behavioral and neuroimaging methodologies. We will discuss the neural substrates of fingerspelling and whether these are shared or distinct from those used to process other forms of communication. We will then consider fingerspelling as it relates to literacy development, discussing overlap with reading pathways as well as brain- behavior correlates that capture associations between fingerspelling and various reading skills. The second goal of this paper is to examine the role of fingerspelling in translanguaging and communication. We will discuss fingerspelling as it relates to translanguaging theories and how it can be leveraged as a translanguaging practice in support of literacy and other communication goals.

## 2. Fingerspelling

### 2.1. Linguistics of Fingerspelling

Fingerspelling is used for a variety of purposes within sign languages, including for proper nouns (e.g., names of people and places), short words (e.g., O-K, S-O, D-U-E, R-I-C-E), acronyms and abbreviations (e.g., for states: ‘Oklahoma’ [O-K-L-A], National Association of the Deaf [N-A-D], and common words such as ‘apartment’ [A-P-T]), for foreign words, for vocabulary unknown to the speaker (even if a corresponding sign exists), for emphasis, for low-frequency words, and to contrast lexical class (as Padden 2005, described: the sign RENT can refer to the verb ‘to rent’, while the fingerspelled word R-E-N-T can refer to the noun referring to the monthly payment, although this pattern may differ based on one’s idiolect) (e.g., Humphries and MacDougall 1999; Padden 2005; Padden and Gunsauls 2003; Schembri and Johnston 2007; Sehyr et al. 2018). The majority of words that are fingerspelled are nouns (both proper and common nouns), followed by adjectives, and then verbs (see Padden and Gunsauls 2003 for ASL; Schembri and Johnston 2007 for Australian Sign Language, Auslan).

Padden (2005) points out that fingerspelling does not exist simply to fill in the gaps in representing English when there is not a direct sign translation; it exists as a natural part of Deaf signers’ productive signing even when there are equivalent signs. Signers sometimes continue to fingerspell even when they could conventionalize a sign for commonly fingerspelled items. For example, ‘diglossia’ has a designated sign in British Sign Language (BSL) but is fingerspelled in ASL (Padden 2005). Fingerspelling handshapes are also used in initialized signs, which use the handshape to denote the first letter of the intended English translation of the sign. Although initialized signs grew in popularity as a means of distinguishing English translations in Manually Coded English, they are now commonly used to distinguish semantically related variants of a natural sign (e.g., the sign for SCIENCE produced with a B handshape for BIOLOGY or a C handshape for CHEMISTRY), which is especially useful for technical vocabulary (Padden 1998). We will not focus on initialized signs in this review, but it is worth noting as yet another example of how fingerspelling serves many different purposes. It is important to recognize that fingerspelling is not just a manual code for spoken language but a naturally acquired, integrated part of the sign lexicon (Padden and Le Master 1985; Sehyr et al. 2018).

**Figure 1.**
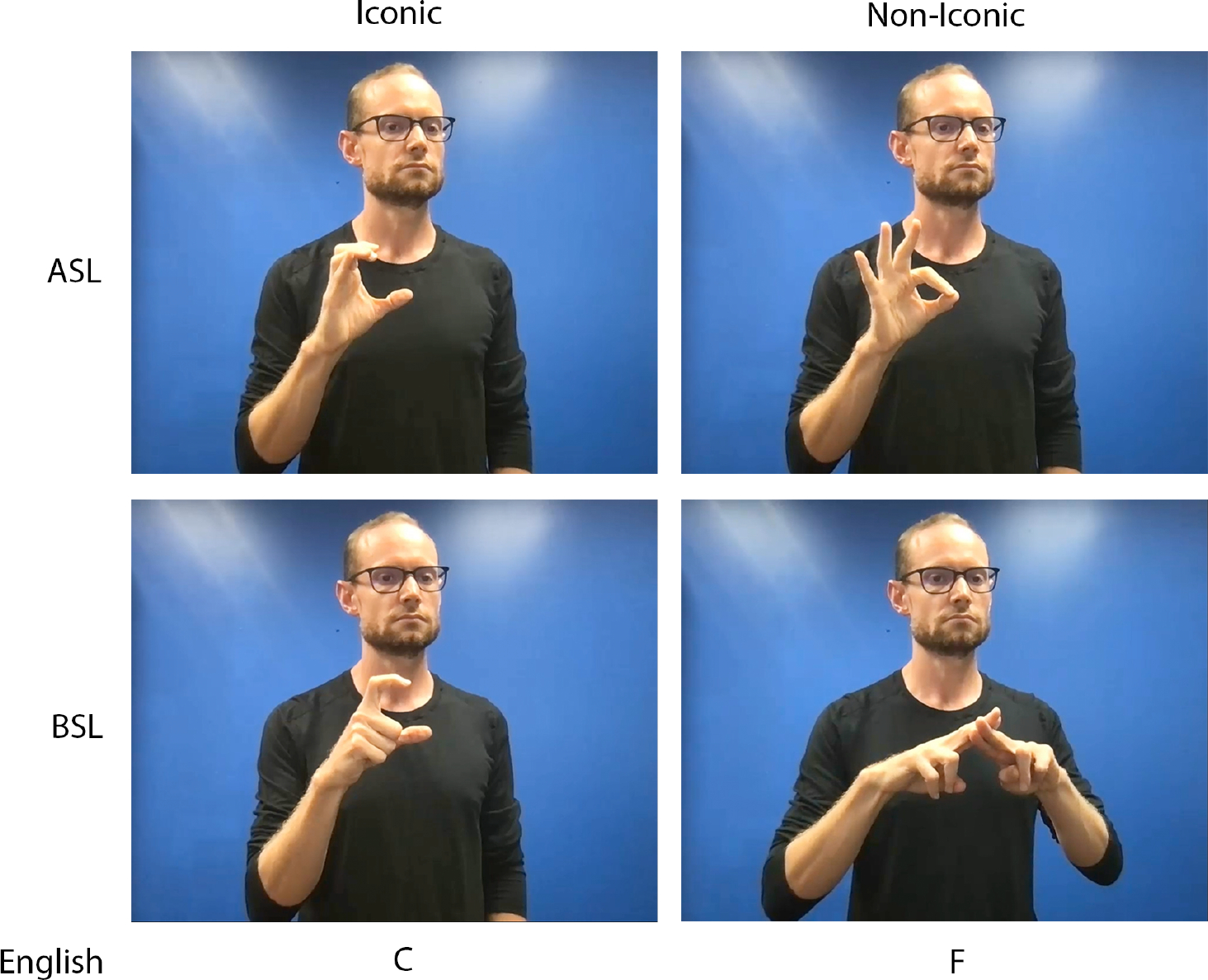
Iconic and Non-Iconic Fingerspelled Letters in ASL and BSL. The iconic fingerspelled letter ‘C’ resembles the English letter ‘C’ in both ASL and BSL. The non-iconic fingerspelled letter ‘F’ does not resemble the English letter ‘F’ in ASL or BSL. ASL fingerspelling is produced with the signer’s dominant hand, while BSL fingerspelling is (mostly) produced with both hands.

Evidence that fingerspelling arises from the signed language rather than the surrounding spoken language can be seen in the fact that BSL and ASL use different handshapes to encode the same English alphabet (see Figure 1), with BSL signers producing fingerspelled letters using both hands while ASL signers use only their dominant hand. Each handshape and orientation configuration in a fingerspelled alphabet corresponds to a written letter, and many handshapes are iconic, or look like the letters they represent (see Figure 1). For example, the handshape configurations of the ASL fingerspelled letter ‘C’ and the BSL fingerspelled letter ‘C’ physically resemble the English letter ‘C’. Other fingerspelled letters are arbitrary, like the handshape for the letter ‘F’ in both BSL and ASL.

Variation in fingerspelling is common across signed languages. Signed languages may use their own unique set of handshapes to represent the orthography of the surrounding spoken language, as is seen with the signed language used by the deaf community in Iran-Zaban Eshareh Irani (Sanjabi et al. 2016). Alternatively, they may use the same or similar fingerspelled alphabet as another signed language but add handshapes for letters not found in the original alphabet, as in Swiss German Sign Language (DSGS), which uses the fingerspelled alphabet from ASL with minor phonetic variations but adds handshapes for ‘ch,’ ‘sch,’ ‘ä,’ ‘ü,’ ‘ö’ (Ebling et al. 2017). For tactile sign languages, fingerspelling mirrors what is used in the corresponding visual sign languages but differs in the modality of reception: Deafblind signers will either place their own hands and palms around the side and back of the signer’s hands or will have the signer produce the fingerspelling directly into their palms (Reed et al. 1990).

Fingerspelling for signed languages where the surrounding community uses a nonalphabetic written language is somewhat different. For example, in Japanese Sign Language, surnames are most frequently “spelled” using two or more signs that correspond to the written kanji characters forming that individual’s name but can also be represented using handshapes representing the kana (or syllabograms) for that name (Nonaka et al. 2015). However, not all sign languages that are surrounded by character-based orthographic systems rely on the same fingerspelling approach. Chinese Sign Language utilizes handshapes to represent the pinyin alphabetic system (26 monosyllabic letters and four disyllabic letters; Jiang et al. 2020). Pinyin is not a direct representation of the character-based written form but rather relies on mastering the letters as well as the “unique representations of onsets, rimes, and tones” in spoken Mandarin (Xiao et al. 2020, p. 2). Nevertheless, pinyin phonetic symbols, pinyin fingerspelling, Chinese Sign Language, and Signed Chinese are used in conjunction with each other in the classroom to support acquisition of literacy skills (Wang and Andrews 2017), an example of how fingerspelling can be incorporated into translanguaging that allows students and teachers to use all of their linguistic and semiotic resources for learning and interacting with each other and the material (Holmström and Schönström 2018).

Different researchers have provided estimates of the amount of fingerspelling that Deaf signers use throughout their daily communication, ranging from 12–35 percent for ASL (Padden and Gunsauls 2003). Fingerspelling appears to be more common in ASL than other sign languages, but its frequency seems to be subject to cross-linguistic variation (Schembri and Johnston 2007; Nicodemus et al. 2017; Beal-Alvarez and Figueroa 2017). For example, Schembri and Johnston (2007) reported that fingerspelling occurred in 10% of lexical items for their sample of Auslan, while McKee and Kennedy (2006) reported that only 2.5% of utterances from New Zealand Sign Language contained fingerspelled items. The amount of fingerspelling used in communication also appears to vary by context (e.g., interviews vs. conversations) and other sociolinguistic factors (such as age or region; Schembri and Johnston 2007).

In addition to the various linguistic and sociocultural factors that lead to variation across fingerspelling systems, there are also unique cognitive, linguistic, and motoric demands for producing and comprehending fingerspelling compared to lexical signs. For a review of the neurobiological foundations of signed language focused on sign production and comprehension with some discussion of fingerspelling see Emmorey (2021). In the sections that follow, we will focus specifically on fingerspelling and how its use and acquisition are similar to and different from those of other types of communication.

### 2.2. Fingerspelling Production

**Figure 2.**
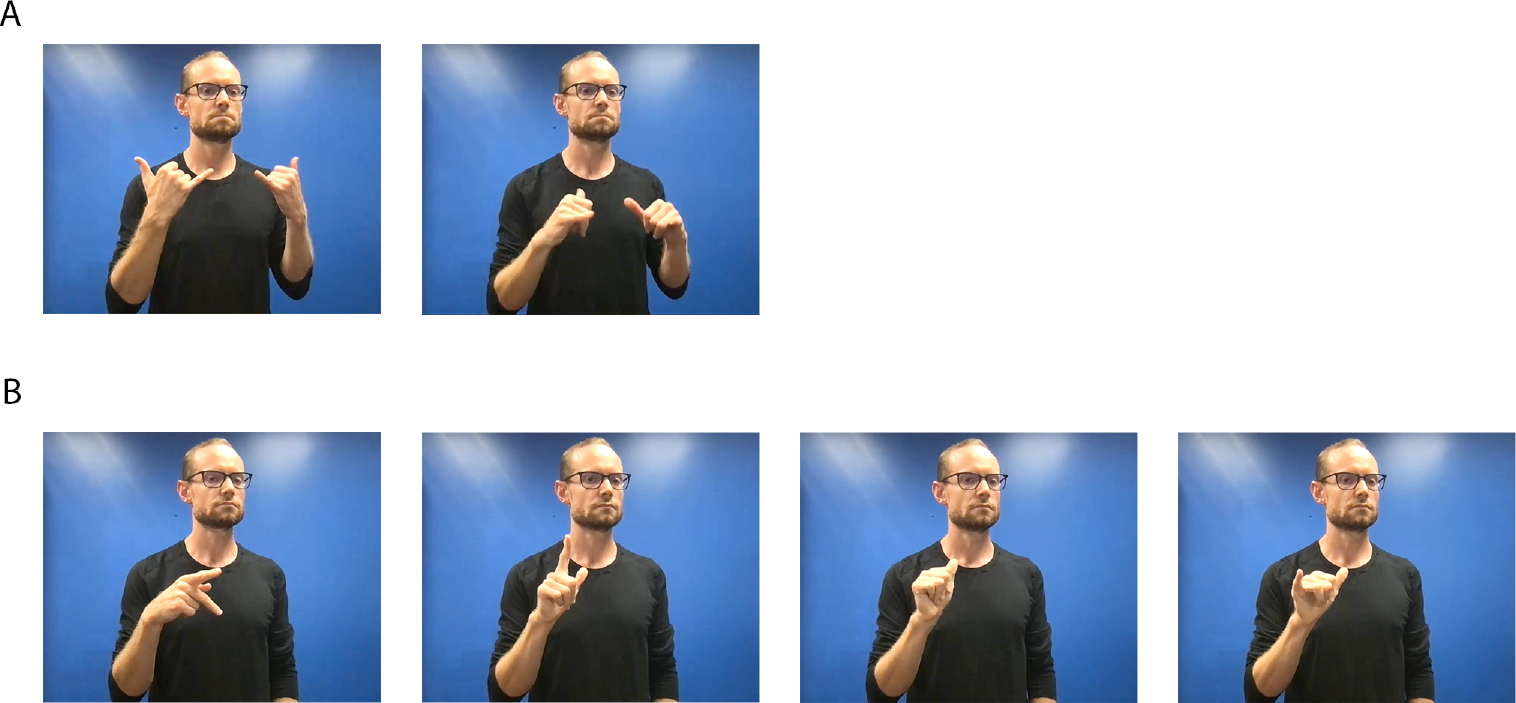
Lexical Sign vs. Fingerspelled Word. (**A**) The lexical sign PLAY in ASL is produced with both hands in a Y handshape and a twisting movement in neutral space. (**B**) The fingerspelled word P-L-A-Y requires four distinct handshapes produced in rapid succession in front of the shoulder of the signer’s dominant hand.

Fingerspelling differs from production of lexical signs in that a sign typically involves one or two handshape changes or location changes, but fingerspelling involves a sequence of rapid changes between complex handshapes that correspond to each letter in a word (see Figure 2). Fingerspelled words therefore take longer to produce than lexical signs and have a later uniqueness point (Waters et al. 2007). Because the handshapes are produced in a fluid continuous motion rather than static holds for each individual letter, coarticulation and simplification are often evident within these complex handshape changes (e.g., Geer and Wilcox 2019; Jerde et al. 2003; Johnston 1998; Lepic 2019; Wilcox 1992). For example, movements from preceding or following handshapes can affect the articulation of a target handshape (Jerde et al. 2003; Keane et al. 2012). During fluent production, signers are not typically aware of each individual letter but rather larger chunks (e.g., the word or word part). The transitions between hand configurations happen rapidly, resulting in approximately 4–5 letters per second although the precise rate varies somewhat by report: Wilcox (1992) reports 4.7 letters per second, while Keane (2014) reports in his 2014 dissertation a rate of 5.84 letters per second. Interestingly, when comparing deaf signers’ productions of fingerspelled words, Wilcox (1992) noticed that some words were produced more fluently, while others were more jerky and less fluent. Compared to less fluent words, the more fluent words had a lower peak velocity (indicating better controlled productions) and less variability in finger movements between repeated productions of the same word. Both metrics indicate that control (and not overall speed) contribute to fluently produced fingerspelling.

Neuroimaging studies underscore the complexity of producing fingerspelled words. In a positron emission tomography (PET) study, Emmorey et al. (2016) found that fingerspelling words engaged ipsilateral motor cortex (including the “hand knob” region responsible for controlling motor movements of the hand and fingers) and cerebellar cortex, which they proposed reflects the complex timing and handshape configurations required to produce ASL fingerspelled sequences compared to one-handed signs. Fingerspelling also showed less activation in left middle temporal cortex compared to signs, likely because fingerspelling a word may not require lexical-semantic processing but translating a word into a sign does.

Fingerspelling also differs from sign production in that the hand’s placement or location is a less salient phonological feature. Fingerspelling is produced with the hand(s) in neutral space. For example, ASL fingerspelling is produced in front of the signer’s dominant shoulder. The hand does not touch other body parts, except the other hand in the case of two-handed fingerspelled letters as in BSL. This neutral placement is reflected in the neural pathways for fingerspelling production. In a PET study by Emmorey et al. (2003), the authors found increased activation of the supramarginal gyrus (SMG) for signs compared to fingerspelled words, which they argued may reflect phonological encoding of place of articulation, which is more important for producing body-anchored signs than fingerspelling in neutral space.

That is not to say that phonological information is not encoded in fingerspelling as well. Although place of articulation may be less relevant, handshape and movement appear to be important phonological parameters that are encoded in the production of fingerspelled words. During a unique situation in which a fluent Deaf signer underwent an awake craniotomy, Leonard et al. (2020) determined that cortical tissue for finger control involved in fingerspelling appeared to be organized into two clear clusters in cortex based on whether handshapes were open (the handshapes for ‘W’/’B’/’C’/’I’) or closed (the handshapes for ‘M’/’T’/’A’/’S’). This distinction between open and closed handshapes reflects the phonological level of language processing. Leonard et al. (2020) further found that postcentral and supramarginal gyri were involved in producing signs and fingerspelling that require lexical processing but not similar types of non-linguistic movements (such as transitional movements). This finding classifies fingerspelling movements as linguistically meaningful, similar to those involved in producing signs but unlike gestures.

Over time, some fingerspelled words become lexicalized so that the phonological parameters of handshape, location, and movement are reduced and the resulting production looks more sign-like (lexicalized signs are typically denoted with #; e.g., Humphries and MacDougall 1999; Valli et al. 2011). For example, #BACK is a commonly used lexicalized sign where the final K is retained but the first three fingerspelled letters have become fused and reduced in movement and handshape realization (see Lepic 2019 and Valli et al. 2011 for further analysis of #BACK). Although these lexicalized fingerspelled words are more standardized, they are not frozen forms and are subject to linguistic pressure to reduce or clearly articulate depending on context (e.g., initial mention of a word requires careful articulation so that comprehenders can identify the word, which is more difficult than simply recognizing a term that has been previously introduced; Lepic 2019).

Overall, although fingerspelling and signs are both manually produced linguistic stimuli, their production differs because fingerspelling involves a rapid sequence of several handshapes and transitional movements between them, whereas signs involve fewer handshapes but are more specified for locations and the movements between them.

### 2.3. Fingerspelling Comprehension

The complex phonological information encoded in fingerspelling during production also presents challenges for perception and comprehension. The visual processing system is more efficient than the auditory system at processing information that is presented simultaneously rather than sequentially, which has ramifications for speech and sign comprehension (e.g., Anobile et al. 2019; Geer 2016; Geraci et al. 2008; Tulving and Lindsay 1967). For example, speech is a series of phonemes that are articulated in rapid sequence (sequential production). Signs on the other hand are produced with the phonological information layered (simultaneous production). Fingerspelling is an instance in which visual information is presented sequentially rather than simultaneously, possibly contributing to the perceived difficulty of processing fingerspelling as opposed to signs. In her dissertation work, Geer (2016) found that explicit instruction about the phonetic components of fingerspelling resulted in improvements in the fingerspelling comprehension scores of second language learners, possibly providing them tools to use in offsetting the mismatch between sequential learning and the visual system.

Signers do not decode each fingerspelled letter. Rather, they “read” the movement contour or movement envelope that is formed by the handshapes and transitions between handshapes rather than each individual handshape (Akamatsu 1982). Accuracy of comprehension is influenced by several factors, including rate of fingerspelled letter presentation, as was found for both visual fingerspelling and tactile fingerspelling using a task requiring correct repetition of fingerspelled sentences (Reed et al. 1990). At typical production rates (approximately 2–6 letters per second), accuracy for both types of fingerspelling comprehension was generally high; however, as the rates sped up, accuracy dropped for both, with the largest variability observed for tactile fingerspelling. Manual similarity of letters also affected accuracy for ASL tactile fingerspelling. For example, the fingerspelled letters ‘A’/’S’/’T’/’N’/’M’/’E’ in tactile ASL were often confused due to the similarity of handshapes created by a lack of extended fingers (Reed et al. 1990). Willoughby et al. (2014) report an example of misunderstanding in Auslan due to the similarity of the handshapes for D-I-M and C-O-M between two skilled users of Tactile Auslan: D and C involve an identical handshape for the dominant hand but differ in whether the dominant hand touches the open palm of the nondominant hand, and I and O involve the index finger of the dominant hand touching the fingertip of the middle finger or the ring finger respectively. In this example, the Deafblind signers utilized translanguaging strategies including signs and fingerspelling to clarify this misunderstanding just as sighted signers do (Willoughby et al. 2014).

Neuroimaging studies have shown that fingerspelling comprehension appears to share some of the same cognitive processes as comprehending lexical signs. In their respective fMRI studies, Waters et al. (2007) and Emmorey et al. (2015) found similar areas of activation in fronto-temporal regions, suggesting shared perceptual networks of activation for fingerspelling and sign comprehension in both BSL and ASL. However, there are also some neurological differences when processing signed and fingerspelled stimuli. Emmorey et al. (2015) found that comprehension of fingerspelling (measured using a semantic judgment task) activated frontal and occipital regions more than did comprehending signs. Furthermore, perceptual processing appears to be aided by top-down linguistic processes when deaf signers comprehend signs but not fingerspelled words presented in the periphery (Schotter et al. 2020).

Perception and comprehension of fingerspelling also show some similarities with processing spoken and written words. Some fingerspelling tasks may engage speech-based phonology. For adult deaf signers who are relatively skilled readers, spoken language phonology appears to influence the recall of fingerspelled words more so than orthographic similarity (Emmorey and Petrich 2012). However, processing fingerspelling also has neural regions in common with processing printed words, as both are visual representations of orthographic information. Emmorey et al. (2015) showed similar cortical areas were activated for comprehension of both print and fingerspelled words (e.g., inferior frontal gyrus, insula, and medial superior frontal gyrus), suggesting both tasks (comprehending fingerspelling and print) recruit neural regions that support processing orthographic information. Activation in the left mid-fusiform gyrus has also been shown for tactile reception of print (i.e., Braille) by blind readers (Büchel et al. 1998). Although future research is necessary in order to determine the neural underpinnings of tactile fingerspelling, it is likely that a similar pattern of activation would be observed for its comprehension due to the orthographic nature of the input, as suggested by Waters et al. (2007). In any case, the left mid-fusiform gyrus appears to play a role in processing orthographic information regardless of the language modality.

Some processing differences for fingerspelling and printed words have also been observed despite both being visual stimuli. Emmorey et al. (2015) found that bilateral visual motion processing regions were more involved in comprehension of fingerspelling than printed words, possibly due to the dynamic nature of fingerspelled words that unfold over time and space in contrast to the static presentation of print. Activation in these regions (inferior parietal lobule, inferior occipital gyrus, middle occipital gyrus) was negatively correlated with participants’ performance on a fingerspelling reproduction task, indicating that the less skilled a person was at reproducing fingerspelling, the more activation was present in these regions when they were processing fingerspelling.

In short, fingerspelling blends features of both sign and print, which is reflected in the neuroimaging and behavioral studies presented above. Fingerspelling comprehension is a challenging but useful skill that appears to recruit some neural systems relevant for both visual stimuli and orthographic information.

### 2.4. Fingerspelling Acquisition

With a better understanding of how adult signers use fingerspelling, we now turn to consider how it is acquired. Deaf children must also learn the intricate processes of comprehending and producing the rapid series of the complex handshapes that comprise fingerspelled words. Children do not initially understand the mapping between printed and fingerspelled letters, and yet they produce fingerspelling before they can read. Specifically, Deaf children who have Deaf parents (DOD) are exposed to fingerspelling from birth as a natural part of their parents’ signing and begin attempting to fingerspell at approximately two years of age. When asked to fingerspell at age two years, nine months, a Deaf child produced at least three handshapes (Padden and Le Master 1985). This timeframe of first producing fingerspelling is corroborated by a case study of a DOD child reported by Blumenthal-Kelly (1995) showing the first productions of fingerspelling to be around 19 to 24 months of age.

Children in the early stages of fingerspelling may not produce fully articulated fingerspelled words. Padden (2005) observed that Deaf children sometimes produce a clear initial handshape and then move their fingers in a manner that mimics the movements seen in fingerspelling without distinctly showing each letter, or they invent a spelling when they do not know the word. This is similar to other reports of young Deaf children initially treating fingerspelled sequences of letters as whole signs rather than individual letters that correspond to print letters (Haptonstall-Nykaza and Schick 2007; Padden 2005; Erting 1999). In fact, Deaf children begin to fingerspell before they have acquired orthographic awareness of the alphabetic principle. At that age, the fingerspelled productions are more holistic in nature, like lexical items where the transitions between the letters are characterized as internal sign movements (Akamatsu 1982). Padden (2005) provides the following example: a DOD child around 24 months old distinguished between the two fingerspelled words I-C-E and R-I-C-E using their movement contours. The former (I-C-E) was produced with a movement of opening the hand (the transition from I to C) followed by a closing movement (the movement C to E), while the latter (R-I-C-E) was produced with a circular movement arising from the influence of the R followed by the other letters.

When Deaf children begin to learn print letters, they go through what Padden termed “the second skill of fingerspelling” (Padden 2005, p. 197). At this point, they are explicitly taught print letters and are starting to make associations with the corresponding fingerspelling handshapes but may still produce the letters in the wrong order (T-E instead of E-T for the alien from the movie E.T.) or the wrong fingerspelling shapes entirely (e.g., spelling the name of a dog U-B-A for ‘Sasha’; Padden and Le Master 1985). Even when children can produce the correct individual fingerspelled letters, they may not yet understand the full relationship between those letters and the meaning of the fingerspelled word. For example, a child at the age of 3 years and 5 months was able to produce the individual fingerspelled letters for a print word shown to her (e.g., R-I-C-E) but was unable to recognize the word until her mother fingerspelled it to her using a more lexicalized form, suggesting that the production of each individual letter had not fully integrated with the representation the child held of the lexicalized word (Blumenthal-Kelly 1995).

Deaf children’s fingerspelling abilities increase significantly from 3 to 5 years of age across a variety of skills including fingerspelling their own name, engaging in “fingerbabbling” (purposeful movements of the fingers that are not accurate fingerspelling handshapes), understanding simple fingerspelled words when produced by someone else, translating written letters into fingerspelled letters, and increasing the number of words they can fingerspell (Allen 2015). Deaf children also increase the number of handshapes they can produce across this age span but in general do not yet produce 50 to 100 fingerspelled words. They are also still learning how to produce fingerspelled words when presented with their ASL translations and how to combine fingerspelling with signs within full sentences (Allen 2015).

A Deaf child’s linguistic environment also affects learning to fingerspell new words. DOD children (both 5- to 6-year-olds and 8- to 9-year-olds) were able to learn to fingerspell common and novel words and nonwords more accurately than Deaf children with hearing parents (DOH) (Hile 2009). However, across both groups (DOD and DOH), the 8- to 9-year-olds were more accurate at producing fingerspelled words than the younger group and showed a strong relationship between performance on a paragraph reading task (in printed English) and receptive vocabulary (administered through a combination of ASL and fingerspelling). Similarly, after students practiced fingerspelling lexicalicalized versions of target words, Haptonstall-Nykaza and Schick (2007) found that DOD children outperformed DOH children on a combined score created from accuracy in fingerspelling production, recognition of print, and writing out a fingerspelled word. Such findings underscore the importance of a child’s language competence and exposure; however, further work is needed in order to determine the contribution of language skills and linguistic environment on fingerspelling development.

Roos (2013) provides a summary of the developmental stages of fingerspelling and literacy generated from a qualitative analysis of six Deaf children (aged 3; 1–6; 9) fluent in Swedish Sign Language (SSL). SSL was their first language, with exposure beginning between 1 and 2 years of age. She describes the following stages of fingerspelling development based on her data:

- Exploring handshapes, letters, and inventing fingerspelling (Age: <4)
- Exploring the direction of writing and fingerspelling (Ages 4–8)
- Practicing and memorizing names, words, and using fingerspelling as a memory aid (Ages 4–8)
- Segmenting fingerspelling words into individuals fingerspelled letters and decoding written words into fingerspelled letters (Ages 4–8)
- Learning the relationships between letters (print and fingerspelled), words, signs, mouth movements, and voice (Ages 4–8)

Although this list was developed from a small sample of Deaf children, this broad picture of the developmental stages is consistent with Padden and Le Master’s (1985) idea of two stages of acquisition for fingerspelling, with the primary stage of a holistic understanding of fingerspelling occurring in the age range prior to 4 years old and the secondary stage where children learn to map orthography to fingerspelling.

Native and non-native signers tend to produce similar error types, including transposition errors (F-A-L-G for F-L-A-G), omissions (G-O-F for G-O-L-F), substitutions (B-R-E-N-D for B-R-E-A-D), and additions (B-A-S-K-E-S-T for B-A-S-K-E-T) (Hile 2009). Deaf children additionally will produce invented spellings for words, where productions do not resemble the target words but are composed of real fingerspelled letters (O-U-M for C-R-A-B). Unsurprisingly, younger children (5–6 years old) were more error-prone than older children (7–8 years old) for both production of fingerspelling and matching fingerspelled words to their sign/picture equivalent (Hile 2009). DOD children made more transposition and substitution errors than DOH children, but DOH children produced more invented spellings. While signing status affected the type of errors the kids made, the frequency of the error types differed based on age: younger children were more likely to produce invented spellings and substitutions than older children (both DOH and DOD groups).

In sum, fingerspelling production begins around two years of age and advances to a holistic stage before the age of four in which preliterate children focus on movement contours as if fingerspelled words were signs. Once a child starts learning to read, they begin to map fingerspelled letters to printed letters so that they integrate fingerspelling into word representations and fluent signing. Reading development clearly plays a role in these later stages of fingerspelling development, and in the next section we will show how fingerspelling also supports reading.

### 2.5. Fingerspelling and Reading

Many studies have now shown a link between fingerspelling ability and literacy skills (Allen 2015; Alawad and Musyoka 2018; Emmorey and Petrich 2012; Harris and Beech 1998; Haptonstall-Nykaza and Schick 2007; Lederberg et al. 2019; Morere and Koo 2012; Ormel et al. 2022a; Padden and Hanson 2000; Padden and Ramsey 1998, 2000; Puente et al. 2006; Sehyr and Emmorey 2022; Stone et al. 2015; Treiman and Hirsh-Pasek 1983; for review see Morere and Roberts 2012). In fact, both Stone and colleagues (2015) and Sehyr and Emmorey (2022) found that fingerspelling accounted for a significant amount of variance in reading abilities of deaf readers, even more so than ASL skill.

While fingerspelling appears to be crucial for reading development, the precise mechanisms of how it supports literacy are unclear. One possibility is that fingerspelling supports phonological awareness. Some studies have linked fingerspelling to speech-based phonology. For example, Sehyr et al. (2017) found that skilled deaf readers used speech-based phonology to retain fingerspelled words in short-term memory. Furthermore, Emmorey and Petrich (2012), found that deaf adult signers produced mouthings associated with English words and pseudowords when fingerspelling, which they interpreted as a stronger link between fingerspelling and phonologically defined syllables than fingerspelling and orthographically defined syllables. In an fMRI study conducted by Dufour et al. (2004), deaf signers showed similar patterns of activation for fingerspelled and printed letters, including bilateral activation of the supramarginal gyrus, a region associated with phonological processing. The authors suggested that signers may have engaged speech-based phonology by silently rehearsing and recruiting phonological working memory when processing fingerspelling for the task. On the other hand, fingerspelling may support reading via sign phonology (Haptonstall-Nykaza and Schick 2007). Through the phonological units of handshape, location, movement, and orientation, signers develop sensitivity to the internal structure of signs and fingerspelling. Lederberg et al. (2019) found that this sign-based phonological awareness is an alternative to speech-based phonological awareness that also supports reading in deaf children. It can even serve as a complement to speech-based phonological awareness for those who have access to both sign and speech.

Besides these ties to phonology, fingerspelling also provides an alternative way to encode orthographic information aside from writing. Orthographic representations may be shared between words and signs; especially since there is no written form of ASL, fingerspelling may strengthen these representations and facilitate language co-activation in sign-print bilinguals (Shook and Marian 2019). Evidence for shared orthographic representations for fingerspelling and print can be seen in the fact that fingerspelling skill correlates with spelling skill (Sehyr and Emmorey 2022) and activates the left mid-fusiform gyrus, known functionally as the Visual Word Form Area (VWFA). The VWFA appears to be specifically recruited for orthographically structured input regardless of whether it is via print (e.g., Dehaene and Cohen 2011), fingerspelling (Emmorey et al. 2015; Waters et al. 2007), or Braille (Reich et al. 2011).

Neural responses to orthographic stimuli may reflect differences in how deaf people learn to read. Several studies have shown that they process orthographic stimuli bilaterally, while language processing classically shows left lateralized activation. In their respective fMRI studies, Dufour et al. (2004) showed no dominant hemisphere of activation for processing fingerspelled letters, and Waters et al. (2007) found that deaf BSL signers showed greater bilateral activation of the visual word form area (VWFA) when they viewed fingerspelled words compared to lexical signs. The authors proposed that the left mid-fusiform gyrus and its right hemisphere homologue aid in mapping visuo-dynamic stimuli like fingerspelling to abstract representations and processing orthographic information regardless of modality.

Bilateral patterns of activation have also been seen in deaf readers’ response to visual word forms. In an ERP study, Sehyr et al. (2020) found less left-lateralization of the N170 in response to words for deaf readers compared to hearing readers. This ERP component is associated with expertise for visual stimuli, and its left-lateralization for words is thought to reflect phonological mapping processes in word reading for hearing people. The fact that deaf people showed greater engagement of the right hemisphere, which was associated with spelling skill, suggests that they may rely more on orthography than phonology when processing visual word forms. Emmorey et al. (2017) also found a more bilateral N170 response to printed words for deaf readers compared to hearing readers, which again correlated with spelling skill. The authors speculated that expertise for print is tuned differently in deaf readers, who rely less on mapping graphemes to phonemes when learning to read. Instead, they may treat words more like visual objects, and their neural tuning to print may involve mapping orthographic information onto words via fingerspelling. Early orthographic learning via fingerspelling is possible for deaf kids as young as preschool, even before they learn to read (Miller et al. 2021). Deaf kids also tend to produce fingerspelling in chunks conforming to syllable segments driven by orthographic units rather than phonological units as seen for hearing kids (Transler et al. 1999) and deaf adults (Emmorey and Petrich 2012). With such an early influence on learning, it is not surprising that fingerspelling might contribute to deaf readers’ unique neural response to printed words. Regardless of the modality and whether based in orthography or phonology, it appears that the ability to segment and manipulate the internal structure of words and/or signs can facilitate various reading skills such as word decoding, word recognition, vocabulary, metalinguistics, reading fluency, and comprehension (Antia et al. 2020; Chamberlain and Mayberry 2000; Haptonstall-Nykaza and Schick 2007; Hirsh-Pasek 1987; Lederberg et al. 2019; Padden and Ramsey 2000; Stone et al. 2015).

The links between fingerspelling and print do not appear to be merely at the sub-lexical level. Sehyr and Emmorey (2022) found that spelling and reading comprehension were more strongly associated with the ability to fingerspell real words compared to pseudowords, indicating a benefit of lexical information and associations built between fingerspelling and word representations. In fact, it has been suggested that deaf readers may have tighter orthographic-to-semantic connections compared to hearing readers (Bélanger and Rayner 2015; Emmorey et al. 2017; Sehyr and Emmorey 2022). Fingerspelled and printed words showed overlapping activation in left middle temporal gyrus and left anterior temporal lobe for deaf signers (Emmorey et al. 2015). The authors suggest that common lexical-semantic networks were engaged for both types of stimuli through orthographic decoding. Since fingerspelling can support lexico-semantic processing, it may be especially useful for word learning. Deaf kids use it to rehearse or memorize words or to introduce unfamiliar words to themselves, producing each fingerspelled letter one-to-one with the printed letters in a word (as a hearing child might read aloud, pronouncing each phoneme), and providing deaf children with models of fingerspelling. In addition to ASL translations, fingerspelling facilitated word learning more than translations alone (Haptonstall-Nykaza and Schick 2007). Thus, fingerspelling can be a powerful tool for vocabulary and reading development.

## 3. Fingerspelling and Translanguaging

### 3.1. Fingerspelling in an Integrated Linguistic System

Fingerspelling is often described as a bridge between words and signs (Crume 2013; Haptonstall-Nykaza and Schick 2007; Tucci et al. 2014), but translanguaging theories provide a different perspective on how an individual may utilize all of their linguistic and semiotic resources to create meaning (e.g., knowledge of print, fingerspelling, signs, gestures, pictures). Rather than conceiving of signed and spoken languages as separate but interactive systems and fingerspelling as a connection between them, translanguaging theories claim that boundaries between languages are arbitrary and instead argue that communicators draw from a single, unified linguistic system depending on their goals (García and Lin 2016; Otheguy et al. 2015). Indeed, evidence has shown that both languages are active during language processing for bimodal (sign-print) bilinguals (Ormel et al. 2022b; Morford et al. 2019). Deaf bimodal bilinguals show neural activation of signs when reading printed words (Meade et al. 2017; Morford et al. 2011) and, to some extent, show activation of words when comprehending signs (e.g., Hosemann et al. 2020; Lee et al. 2019). These patterns of activation-even when the task specifically calls for responding in only one language/modality-provide additional support for this key translanguaging idea: that speakers are not monoglossic with two separate linguistic systems but heteroglossic with an integrated linguistic system (García and Lin 2016). As reviewed above, fingerspelling has clear cognitive and linguistic ties to signs, spoken words, and printed words, but further consideration of the role fingerspelling plays in such an integrated system is warranted.

Translanguaging theories challenge traditional views of multilingualism and state that named languages like ASL and English are social constructs. A language (such as Spanish) is not a clearly demarcated object that is separate from another language (such as Portuguese) except through political and cultural definitions (e.g., Otheguy et al. 2015; Wei 2018). Fingerspelling is traditionally viewed as an integrated part of signed languages and therefore belonging to deaf communities, perhaps to protect it as part of a minoritized language and Deaf culture. From a translanguaging perspective, however, the distinctions between languages are arbitrary rather than inclusive of an individual’s full range of communicative abilities. Some proponents of translanguaging call for a softening of the boundaries between languages and might argue that fingerspelling belongs to all who include it in their linguistic repertoire. Critics of translanguaging theories argue that they undermine minoritized communities and endanger their languages (e.g., Lyster 2019). For example, the use of some translanguaging strategies in deaf education have been criticized for their limited sensorial accessibility, prioritization of spoken languages, and threat to sign languages as minoritized languages (De Meulder et al. 2019). Multimodal communication may be appropriate depending on the setting and necessary to accommodate non-signing communication partners but risks creating a false equivalence between communication systems (e.g., Manually Coded English, Simultaneous Communication) and language (e.g., ASL). Misapplication of translanguaging strategies could therefore undermine long- and hard-fought efforts to validate signed languages as natural languages (e.g., Bellugi and Fischer 1972) and may increase risk of language deprivation by implying that other forms of communication will suffice in place of language (e.g., Scott and Henner 2021). However, translanguaging advocates claim to support the same goal of protecting minoritized languages by disrupting the social hierarchy that suppresses them in the first place (García and Lin 2016; Otheguy et al. 2015). We assert that fingerspelling is a remarkable translanguaging tool because of its unique status: it is a naturally acquired and fully accessible part of many signed languages that supports multimodal and multilingual communication and can be leveraged during communication exchanges with a variety of interlocutors (both hearing and deaf) in order to create meaning.

### 3.2. Inside and Outside Perspectives on Fingerspelling

Otheguy et al. (2015) discussed what they term the inside and outside perspectives of translanguaging. The inside perspective refers to the cognitive processes that integrate linguistic resources to make meaning. Fingerspelling is often used for non-communicative purposes such as learning, rehearsing, problem solving, etc. (Haptonstall-Nykaza and Schick 2007; Miller et al. 2021; Roos 2013). For example, a deaf student may fingerspell a word under their desk to work out its spelling for a written test. Fingerspelling can be utilized by students in the classroom when practicing or memorizing words (similar to a hearing child who is pronouncing each sound for a word aloud), and as Haptonstall-Nykaza and Schick (2007) showed, providing children with both the fingerspelling and the sign facilitated better learning of the word than the sign alone. In these examples, the signer uses fingerspelling to construct meaning for their own cognitive benefit rather than directing it towards an interlocutor for communication.

On the other hand, the outside perspective states that communicators learn to separate their languages through formal instruction and social interactions (Otheguy et al. 2015). An individual’s internal experience of language blends all of their structural and lexical components into their idiolect, but they select the features of their language system that they wish to employ in any given situation. Proponents of these theories argue that the speaker considers their audience’s idiolect when selecting which structural or lexical features to utilize. Adapting one’s signing and fingerspelling to accommodate a communication partner exemplifies this socially driven feature selection process. Just as monolinguals can vary their linguistic production (e.g., switching registers or speaking differently to a child than an adult), so too fingerspelling can be used for a variety of social and communicative purposes. Fingerspelling is often used if the interlocutor does not know a specific sign, for emphasis, for clarifying a misunderstanding, to show connections to print letters, or to introduce new vocabulary. Signers might also adjust their fingerspelling productions depending on their perceived skill level of the communication partner or on shared background, and there are differences in signing and fingerspelling between deaf-hearing interlocutors and deaf-deaf interlocutors (e.g., Lucas and Valli 1992). For instance, Hein (2013) described how a deaf teenaged student added fingerspelling after using a sign they were not sure was known to their hearing interlocutor as a method to ensure comprehension. Deaf adults tend to use fingerspelling more within their signing when conversing with a hearing adult rather than when conversing with another Deaf adult (Padden and Le Master 1985), and less skilled second language learners of a signed language (M2L2, in reference to their second modality and second language) rely more on fingerspelling than more skilled learners (Leeson et al. 2020). Fingerspelled words are likely to be produced quickly or reduced in form if both interlocutors share a specialized vocabulary, similar to the examples presented by Lepic (2019).

This flexibility is one the reasons why fingerspelling is such powerful translanguaging strategy: signers and comprehenders from a variety of backgrounds, language proficiencies, and contexts can leverage fingerspelling during interactions in order to construct meaning. It is a relatively constrained set of productions that can be learned and then used to convey any number of words or concepts as opposed to the number of individual signs and predicates that would be required to produce the same content. While producing and comprehending fingerspelling is relatively more costly than producing individual signs, its versatility makes it a unique asset that signers have at their disposal.

Applying these translanguaging perspectives to fingerspelling highlights its versatility as both a cognitive and communicative tool. Teachers should acknowledge and make space for both inside and outside perspectives so that fingerspelling can be used to support internal cognitive processes involved with making meaning as well as social and communicative processes to co-construct meaning with interlocutors. In the section that follows, we will expand on educational implications of adopting a translanguaging perspective in the classroom.

### 3.3. Translanguaging in the Classroom

Because languages are considered social constructs within translanguaging theories, the concepts of ‘monolingualism’ and ‘bilingualism’ are appropriate for considering sociolinguistic aspects of language but not lexical or structural ones (Otheguy et al. 2015). This carries significant implications for the education and assessment of bimodal bilinguals. Monolingual approaches to teaching and assessment are not fair or appropriate for these students. For example, excluding fingerspelled responses from lexical assessments would unfairly penalize deaf students, giving an inaccurate picture of their skills, especially when compared to monolingual standards (Sehyr et al. 2018). Rather than building and assessing proficiency in separate named languages in multilingual learners, Canagarajah (2011) favors developing and testing communicative competence, or the student’s ability to negotiate meaning across and between languages for a variety of linguistic functions, interlocutors, and communicative situations. Instead of focusing on transfer errors that interfere with students’ English writing, for example, a translanguaging perspective recognizes that multilingual students have a unique writing process that makes use of both of their languages or their entire unified linguistic system (Velasco and García 2014; Hornberger and Link 2012). Although there is no written form of ASL, for example, bimodal bilinguals may engage in multiliteracy by gathering their thoughts in sign or rehearsing spelling through fingerspelling.

Deaf people are already skilled and experienced in multimodal, translingual communication (Kusters 2017). They often utilize some combination of signed and spoken languages; learned systems like writing, Total Communication, Simultaneous Communication, Manually Coded English, Cued Speech, and speech reading; and nonverbal communication like facial expressions and gestures. In a flexible discursive space unrestricted by monolingual norms, students and teachers are free to make use of all communicative options available to them. Holmström and Schönström (2018) described several trans-languaging practices used by three deaf lecturers as pedagogical and communication tools. These lecturers taught entirely in Swedish Sign Language (SSL) at a higher education institute where both deaf and hearing students were enrolled. The deaf instructors utilized SSL sign language for classroom instruction and discussions, as well as written Swedish and English, enactments, fingerspelling, and mouthing. They also made use of white boards, smart boards, paper, books, and computers to display diagrams, text, images, and videos in order to ensure comprehension. The instructors used fingerspelling to spell out central vocabulary and concepts as well as word parts (such as ‘phonotactics’). This translanguaging process allowed for the instructors to discuss phenomena from other languages (such as Swedish or English) within SSL, engaging students’ understanding across each of those systems and modalities. Wang and Andrews (2017) similarly describe use of fingerspelling alongside signs, print, pictures, and other tools in an educational setting to help deaf children comprehend academic concepts and new vocabulary. Bilingual and bicultural classrooms allow for dynamic languaging more freely, as teachers and students share more of the same words in their idiolects. In these spaces, communicators are able to select more features of their language system and also employ the numerous other ways in which deaf people engage in multimodal translanguaging.

**Figure 3.**
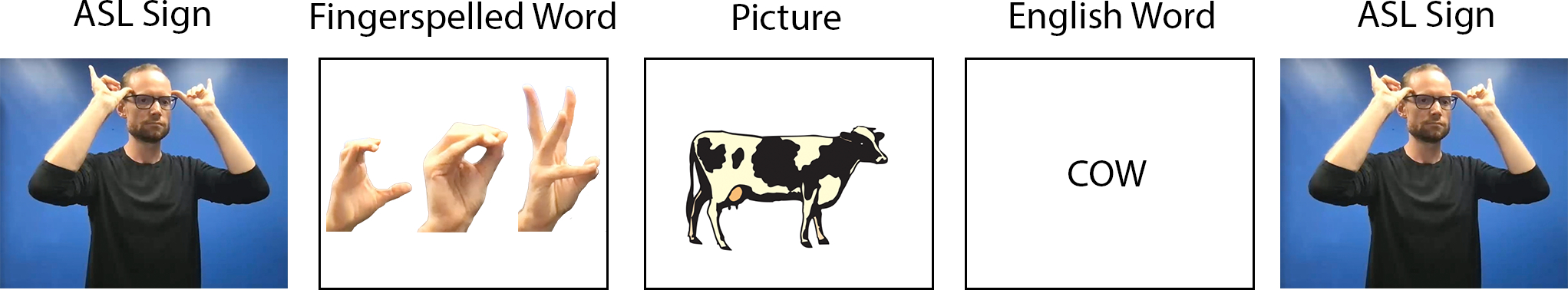
Example of Chaining. The instructor produces a chain including the ASL sign for COW, the fingerspelled word, a picture of the concept, the printed word, and the sign again.

Teachers should not only make space for translanguaging in the classroom but also model and reinforce dynamic translanguaging practices in a multimodal, multilingual setting. Deaf children need to be provided with explicit instruction and modeling of the complex relationships between different forms of communication within an integrated system. Fingerspelling is a key component of several teaching practices commonly used in deaf education like chaining, in which an instructor produces a sign immediately followed by the fingerspelled word, the printed word, and/or a picture followed by the sign again (see Figure 3; Humphries and MacDougall 1999). Fingerspelling and print are both important avenues in which Deaf people are exposed to new English vocabulary and can be highly effective when combined with sign language such as in chaining (Morere and Roberts 2012). Additionally, deaf instructors utilize fingerspelling within chains as a part of their translanguaging practice (Holmström and Schönström 2018). Another common practice is sandwiching, in which an instructor produces a sign followed by the fingerspelled equivalent and then the sign again (e.g., the sign for HAT-the fingerspelled letters H-A-T and then the sign for HAT). These strategies help build associations between a fingerspelled word and other representations of the same concept to help support skilled reading.

Deaf teachers of Deaf students (TODs) seem to have an intuitive understanding that fingerspelling should be used to support students in the process of making meaning. In one study looking at the teaching practices of TODs, Deaf TODs produced, on average, more instances of chaining than hearing TODs (30 vs. 5.5 instances; Humphries and MacDougall 1999). Deaf parents additionally understand that deaf children need to be exposed to fingerspelling as a natural part of communication from birth rather than waiting for them to learn the alphabetic principle. For example, Blumenthal-Kelly (1995) reported that the Deaf parents of the single case study participant produced fingerspelling to their Deaf child when the child was as young as 8 weeks old, and possibly earlier but it was simply not captured by the video camera. Morere and Koo (2012) also advocate for early and frequent use of fingerspelling because of its correlation with various ASL and English skills as well as a number of more general measures including memory, literacy, and academic achievement.

While it is clear that fingerspelling adds cognitive, linguistic, and social value to a student’s translanguaging practices, many hearing TODs and hearing parents of Deaf children experience ‘lexidactylophobia’, or the avoidance (fear) of fingerspelling (Grushkin 1998). Given the complexity of producing, comprehending, and acquiring fingerspelling that we have discussed above, it is understandable that some parents and professionals, many of whom are hearing non-native signers, may have difficulty mastering this skill and are reluctant to model it for their children. Fingerspelling is an especially difficult skill for second language learners to acquire (Geer and Keane 2018), and most learners report that it is easier to produce than comprehend fingerspelling (Șerban and Tufar 2020; Wilcox 1992). M2L2 signers tend to rely heavily on fingerspelling during initial phases of acquisition and then reduce the frequency of fingerspelling as their language competence grows (Leeson et al. 2020). These signers can benefit from evidence-based instruction in fingerspelling for their own communicative competence and as stakeholders in deaf education. The educational curriculum both for Deaf children learning their first language as well as M2L2 learners must prepare students to incorporate fingerspelling in their linguistic repertoires. Below we offer some suggestions for optimizing fingerspelling instruction based on the evidence reviewed here:

Some TODs tend to model fingerspelling one letter at a time even though evidence suggests that showing the entire fingerspelled word is more effective in learning those words (Roos 2013). Therefore, being sure to provide the entire fingerspelled word, including its movement envelope, would be more beneficial for children when learning to fingerspell than producing fingerspelled letters at the single letter level. Including lexicalized versions of fingerspelled words (i.e., adding movement envelopes that make the fingerspelling resemble the related sign more closely) in addition to using the sign and printed word can be more helpful for retaining new vocabulary terms than chains that include the sign and word without fingerspelling (Haptonstall-Nykaza and Schick 2007). Additionally, evidence shows that Deaf adults who are skilled readers rely more on phonological syllables (often coupled with mouthing) when producing fingerspelled words than orthographic syllables (Emmorey and Petrich 2012). This relationship suggests that producing mouthing consistent with the spoken language phonology of a word while fingerspelling may be beneficial when teaching deaf children. Similarly, including spoken words in chains would be appropriate for deaf children who can access both sign and speech and can benefit from phonological awareness in both modalities (Lederberg et al. 2019).

Fingerspelling instruction should be inclusive of diverse sign models due to the idiolectical and phonetic variation seen between communicators. Geer and Keane (2018) found that instruction in the variations seen in different articulatory realizations of the handshapes in fingerspelling can help M2L2 learners comprehend fingerspelling. Such comments were echoed by feedback from participants in Thoryk’s (2010) investigation of a packaged fingerspelling program. The participants indicated that fingerspelling looks different across producers and suggested that having a variety of sign models in the curriculum would be beneficial during the learning process. Learning from diverse signers can increase fingerspelling proficiency as well as accessibility. For example, in Swedish Sign Language, ‘lake’ and ‘ocean’ are homophones that can be distinguished by mouthing differences. The two signs are also indistinguishable in Tactile Swedish Sign Language because a Deafblind signer would not have access to those visual mouthing cues. Instead, Deafblind signers use fingerspelling to disambiguate between the two signs (Mesch 2001). Providing children with models of different ways in which translanguaging can be utilized depending on the context (e.g., frequency and appropriateness of fingerspelling) can help them to learn these situational differences in communication styles as well as encourage their own translanguaging practices. Deaf children must be shown this process of utilizing their whole language system in order to help them communicate more effectively, and in this case as in others, fingerspelling is the conduit for successful communication.

In sum, the evidence base for fingerspelling can inform the instructional materials and practices used to build proficiency in fingerspelling as a discrete linguistic skill and integrate it into translanguaging practice for broader communicative competence.

## 4. Conclusions

Fingerspelling is often overlooked or mentioned as an aside in reports of deaf education pedagogy and sign language studies. In this review, we have highlighted its complexity as a cognitive-linguistic phenomenon and its utility as a naturally acquired linguistic skill that fulfills a multitude of cognitive and communicative purposes. We argue that this visuo-manual orthographic code blurs the lines between signed, spoken, and written languages, making it an essential component of successful translanguaging for Deaf individuals. We hope that this review provides a foundational understanding and inspires further investigation of fingerspelling and its role in translanguaging for multilingual and multimodal communicators.

## Data Availability

Not applicable.
